# Single short reprogramming early in life increases healthspan

**DOI:** 10.18632/aging.204457

**Published:** 2022-12-26

**Authors:** Ollivier Milhavet, Jean-Marc Lemaitre

**Affiliations:** 1 IRMB, Univ Montpellier, INSERM, Montpellier, France; 2 IRMB, Univ Montpellier, INSERM, CNRS, Montpellier, France

**Keywords:** aging, epigenetics, longevity, healthspan, transient reprogramming

Aging leads to a progressive decline of the physiological functions of the organism which leads to an increase occurrence of age-related pathologies like cancer, cardiovascular disorders, diabetes, atherosclerosis, age-related macular degeneration or neurodegeneration ultimately precipitating death. It severely increases the probability of disabilities and consequently affects healthspan. The molecular and cellular processes that governs the mechanisms of aging remain poorly understood but are unambiguously associated to a decreased regenerative capacity that intensifies cell and tissue vulnerability and promotes deterioration with time. However, current knowledge makes it difficult to develop prophylactic strategies to increase healthspan if not lifespan.

Among the multiple possible strategies emerged approaches based on cellular reprogramming. Indeed, the discovery that adult somatic cells could be converted into pluripotent cells (known as induced pluripotent stem cells or iPSCs) was a major breakthrough that motivated the Karolinska Institute in Sweden to select Shinya Yamanaka and Sir John Gurdon as the recipients of the Nobel Prize in Physiology and Medicine in 2012. Technically, when overexpressed in mice or human cells, four transcription factors OCT4, SOX2, KLF4, C-MYC (OSKM) induce a global remodeling of epigenetic landscape to revert cell identity into a pluripotent embryonic-like state [1]. Nevertheless, in the early days of cellular reprogramming, cellular senescence and aging were strikingly described as a barrier to iPSC generation. However, we overcame this obstacle using an optimized strategy to derive iPSCs from senescent cells and cells from centenarians’ individuals using two additional factors NANOG and LIN28 [2]. Ultimately, these redifferentiated cells proved to be rejuvenated, demonstrated the reversibility of cellular aging.

Translating these discoveries into efficient rejuvenating strategies at the organismal level was not straightforward since at first *in vivo* OSKM expression in mice models was described to generate teratomas leading to death [3]. Thus, we and others, speculated on the hypothesis that triggering the reprogramming process *in vivo* and stopping it before obtaining pluripotent cells could allow to dissociate dedifferentiation from rejuvenation. In erasing hallmarks of cellular aging without changing the cell identity, we expected to eventually apply it *in vivo* to improve tissue regeneration in order to have a positive impact on age-related pathologies. Using a mouse model of premature aging Ocampo et al. first validated this hypothesis by inducing cyclic expression of OSKM factors two days a week under the control of doxycycline during the entire life in a process that could involve epigenetic remodeling during cellular reprogramming [4]. The treated mice presented an increased longevity as well as amelioration of tissue regeneration after experimentally induced injury. This paradigm was confirmed in human cells by expression of mRNAs to promote amelioration of cellular aging by transient cellular reprogramming without altering cell identity [5]. However, how OSKM induction might increase lifespan in preventing tissues aging and age-related diseases is still an unresolved issue.

Among the hypothesis raised to decipher the rejuvenation mechanism, epigenetic remodeling has been consistently proposed as the main rejuvenation driver although modulation of adult stem cell plasticity cannot be excluded. Ocampo et al in their seminal study found a rearrangement of histone marks H3K9me3 and H4K20me3, following transient reprogramming, known to be impacted by aging and an increase in the number of Pax7+ satellite cells, which are the muscle stem cells involved in fiber regeneration was observed when they analyzed the capacity of the muscle to regenerate. More recently, studies focusing on DNA methylation profiles have shown that short-term partial reprogramming results only in minor epigenetic DNA methylation modification and in very few organs [6, 7]. DNA methylation clocks, a well-recognized marker of aging, revealed no reduction of the calculated epigenetic age in short-term partially reprogrammed animals while a reduction of the biological age was only observed in some tissues (i.e., kidney and skin) of long-term partially reprogrammed animals [6]. In a parallel study, using a different reprogrammable mouse model, a short expression of the Yamanaka’s factors for 1 week led to DNA methylation changes in regions associated with aging but this was associated mainly to very old animals (100 weeks) in only some organs (pancreas, spleen and liver) and not associated to histological changes that could be correlated to a physiological impact [7].

**Figure 1:**
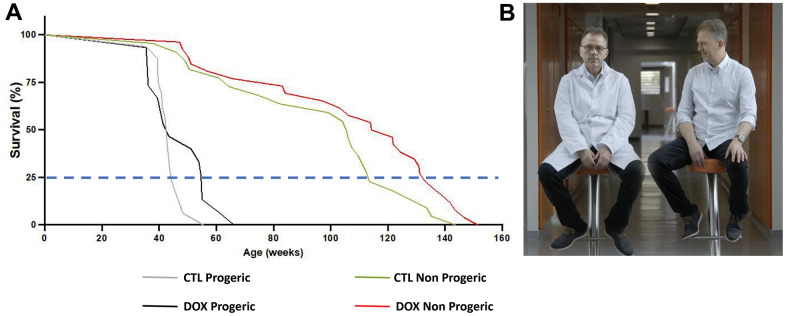
(**A**) Summary of recent findings in Alle et al. [8]. In progeric reprogrammable mouse the lifespan for the third quartile increased by 23.7% for treated animals (DOX) compared to controls (CTL). In non progeric reprogrammable animals, a similar effect was observed with a 16.8% increase for the third quartile. (**B**) Illustration of what it could be imagined for humans. On the left a 60 years old Dr. Jean-Marc Lemaitre has been rejuvenated to around 50 years old on the right. Courtesy of Arte TV Network ARTE Film "In search of lost youth" produced by Galaxie presse in 2022 and directed by Sylvie Gilman and Thierry De Lestrades.

Recently, we have reevaluated and tweaked these rejuvenating paradigms in reprogrammable heterozygous progeroid mice by inducing expression of the 4 reprogramming factors in the whole organism [8]. We showed that, a single short treatment for 2.5 weeks, applied early in life had a protective effect on tissue deterioration at the onset of age-related pathologies. Indeed, in progeric reprogrammable mice, body composition was improved as demonstrated by the higher lean mass proportion associated with a lower fat mass proportion and, concomitantly, motor skills were maintained during aging. We also demonstrated that this short transient reprograming protocol in mouse early life ameliorates age-related condition state of organs such as bone, lung, spleen and kidney as well as skin structure that latter tissue being tremendously positively impacted. In order to address the rejuvenating mechanism, we focused on CpG DNA methylation associated with aging progression in treated and untreated animals. Strikingly, our short reprogramming protocol was able to modify in a tissue-specific manner some CpG modifications counteracting the epigenetic drift associated with aging. Our results indicates that a single short reprogramming early in life might initiate and propagate an epigenetically related mechanism to promote a healthy lifespan. In addition, we established that our enhanced protocol ameliorated the lifespan of aged animals since the age of death for the third quartile increased by 23.7% for treated animals compared to controls (Figure 1A). Most importantly, a similar induction protocol for just two and a half weeks at 2 months of age in non-progeric mice led to a similar increased (16.8%) lifespan in old age (Figure 1A). Of note; it was absolutely remarkable to observe that a treatment at 2 months of age could have consequences around 2.5 years later demonstrating a deep and persistent effect, suggesting a memory effect of transient reprogramming on healthspan, likely driven by an epigenetic process. Our results indicated for the first time that a single short transient expression of reprogramming factors *in vivo* may increase lifespan in naturally aging mice. If we try to imagine, with all the necessary cautiousness, the consequences that this could have on humans, a basic rule of 3 would lead us to consider that a human being entering the third quartile of his life at 60 years old would be as healthy as one of 50 years old that is far from being negligible in terms of life quality increasing potentially his healthy lifespan of around 10 years (Figure 1B).

Our study adds to the body of evidence that allows to consider transient reprogramming as a future strategy for improving healthy aging. It could be argued that most of the results published were obtained in transgenic animals recapitulating accelerated aging raising doubts about the feasibility of such an approach. However, our demonstration in non-progeric animals, although requiring further analysis, indisputably demonstrates, in our opinion, that this strategy offers great promise to treat aging as a disease in a preventive manner with the consequence of reducing the impact of age-related diseases and thus promoting healthy aging.
